# Supplementary material for: Tumor Response, Disease Control, and Progression-Free Survival as Surrogate Endpoints in Trials Evaluating Immune Checkpoint Inhibitors in Advanced Non-Small Cell Lung Cancer: Study- and Patient-Level Analyses
Source: Cancers (Basel). 2022 Dec 28;15(1):185. doi: 10.3390/cancers15010185 (PMC9818635; doi:10.3390/cancers15010185)
Supplement: Supplementary file 1 [file cancers-15-00185-s001.zip › cancers-2018440-supplementary.pdf]

Tumor response, disease control, and progression-free survival as surrogate endpoints in trials evaluating immune checkpoint inhibitors in advanced non-small cell lung cancer: study- and patient-level analyses

Author: Nobuyuki Horita, MD, PhD

Supplementary Materials

Table S1. Formulas used in database searches

Table S2. List of studies used for independent-patient-data analysis

Figure S1. Scatter plot of overall survival and progression-free survival using independent-patient-data

Figure S2. Preferred Reporting Items for Systematic Reviews and Meta-Analyses flow diagram for the trial-level analysis

Figure S3. Sample data used for Monte Carlo simulation

Figure S4. Monte-Carlo simulation for reciprocal duplication

Reference S1. Review articles used in hand search

Reference S2. Original articles for trials used for the study-level analysis

Table S1. Formulas used in database searches.

PubMed, 175 articles
**#1** (non-small cell lung cancer [title] OR non-small-cell lung cancer [title] OR non-small-cell lung carcinoma [title] OR NSCLC [title] OR squamous lung [title] OR adenocarcinoma lung [title])<br><br>**#2** (randomized[title] OR randomised [title] OR RCT [title] OR phase III [title] OR phase 3 [title] OR randomly OR N Engl J Med)<br><br>**#3** (ICI [title] OR immune checkpoint inhibitor [title] OR Nivolumab [title] OR Pembrolizumab [title] OR Spartalizumab [title] OR Cemiplimab [title] OR Avelumab [title] OR Atezolizumab [title] OR Durvalumab [title] OR Ipilimumab [title] OR Tremelimumab [title] OR Camrelizumab [title] OR Sintilimab [title] OR Sugemalimab[title])<br><br>**#4** (non-small cell lung cancer OR non-small-cell lung cancer OR non-small cell lung carcinoma OR NSCLC OR squamous lung OR adenocarcinoma lung)<br><br>**#5** (randomized OR randomised OR RCT OR phase III OR phase 3 OR randomly OR N Engl J Med)<br><br>**#6** (ICI OR immune checkpoint inhibitor OR Nivolumab OR Pembrolizumab OR Spartalizumab OR Cemiplimab OR Avelumab OR Atezolizumab OR Durvalumab OR Ipilimumab OR Tremelimumab OR Camrelizumab OR Sintilimab OR SugemalimabOR Checkmate OR Keynote OR IMpower)<br><br>**#7** HR OR hazard ratio OR cox hazard<br><br>**#8** ((#1 AND #2 AND #6) OR (#4 AND #2 AND #3) OR (#1 AND #5 AND #3)) and #7
Web of Science, 230 articles
**#1** TI=(non-small cell lung cancer OR non-small-cell lung cancer OR non-small-cell lung carcinoma OR NSCLC OR squamous lung OR adenocarcinoma lung)<br><br>**#2** TI=(randomized OR randomised OR RCT OR phase III OR phase 3) OR TS=(randomly)<br><br>**#3** TI=(ICI OR immune checkpoint inhibitor OR Nivolumab OR Pembrolizumab OR Spartalizumab OR Cemiplimab OR Avelumab OR Atezolizumab OR Durvalumab OR Ipilimumab OR Tremelimumab OR Camrelizumab OR Sintilimab OR Sugemalimab)<br><br>**#4** TS=(non-small cell lung cancer OR non-small-cell lung cancer OR non-small-cell lung carcinoma OR NSCLC OR squamous lung OR adenocarcinoma lung)

**#5** TS=(randomized OR randomised OR RCT OR phase III OR phase 3 OR randomly)<br><br>**#6** TS=(ICI OR immune checkpoint inhibitor OR Nivolumab OR Pembrolizumab OR Spartalizumab OR Cemiplimab OR Avelumab OR Atezolizumab OR Durvalumab OR Ipilimumab OR Tremelimumab OR Camrelizumab OR Sintilimab OR Sugemalimab)<br><br>**#7** TS=(HR OR hazard ratio OR cox hazard)<br><br>**#8** ((#1 AND #2 AND #6) OR (#4 AND #2 AND #3) OR (#1 AND #5 AND #3)) and #7
Cochrane CENTRAL, 338 articles
**Combine all of the following using AND**<br><br>**Record Title:** non-small cell lung cancer OR non-small-cell lung cancer OR non-small-cell lung carcinoma OR NSCLC OR squamous lung OR adenocarcinoma lung<br><br>**Record Title:** ICI OR immune checkpoint inhibitor OR Nivolumab OR Pembrolizumab OR Spartalizumab OR Cemiplimab OR Avelumab OR Atezolizumab OR Durvalumab OR Ipilimumab OR Tremelimumab OR Camrelizumab OR Sintilimab OR Sugemalimab<br><br>**Abstract:** HR OR hazard ratio OR cox hazard
EMBASE, 80 articles
ti(non-small cell lung cancer OR non-small-cell lung cancer OR non-small-cell lung carcinoma OR NSCLC OR squamous lung OR adenocarcinoma lung) AND ti(randomized OR randomised OR phase III OR phase 3)<br><br>**AND**<br><br>ti(ICI OR immune checkpoint inhibitor OR Nivolumab OR Pembrolizumab OR Spartalizumab OR Cemiplimab OR Avelumab OR Atezolizumab OR Durvalumab OR Ipilimumab OR Tremelimumab OR Camrelizumab OR Sintilimab OR Sugemalimab OR Checkmate OR Keynote OR IMpower) **AND**<br><br>(hazard ratio OR cox hazard)

Table S2. List of studies used for independent-patient-data analysis.

	Country	Phase	Patho	Stage	PD-L1 status	Driver	PS	Line	Imaging evaluation	Treatment	ROB H/U/L
Fehrenbacher (2016) POPLAR	USA	II	NSCLC	Adv, Met	Any	Any	0-1	2-3	RECIST	Atz (1200 mg) q3w<br>Dtx (75 mg/m2) q3w	2/0/4
Jotte (2020) IMpower131	USA	III	SQ	IV	Any	Any	0-1	1	RECIST	Atz (1200 mg) + Cbdca (AUC 6) + nPtx (100 mg/m2) q3w<br>Cbdca (AUC 6) + nPtx (100 mg/m2) q3w	1/0/5
Peters (2017) BIRCH	Spain	II	NSCLC	IIIb, IV	IC>5% or TC>5%	Any	0-1	1	RECIST ICR	Sigle-arm study<br>Atz (1200 mg) q3w	NA
Rittmeyer (2017) OAK	USA	III	NSCLC	IIIb, IV	IC>1% or TC>1%	Any	0-1	2-3	RECIST	Atz (1200 mg)<br>Dtx	2/0/4
Socinski (2018) IMpower150	Germany	III	NSQ	IV, Rec	Any	EGFR(-), ALK(-)	0-1	1	RECIST ICR	Atz (1200 mg) + Cbdca (AUC 6) + Ptx (200 mg/m2)<br>Atz (1200 mg) + Bev (15 mg/kg) + Cbdca (AUC 6) + Ptx (200 mg/m2)<br>Bev (15 mg/kg) + Cbdca (AUC 6) + Ptx (200 mg/m2)	1/0/5
Spigel (2018) FIR	USA	II	NSCLC	Adv	IC>5% or TC>5%	Any	0-1	Any, no Plat for 6 months	RECIST	Sigle-arm study<br>Atz (1200 mg) q3w	NA
West (2019) IMpower130	Italy	III	NSQ	IV	Any	EGFR(-), ALK(-)	0-1	1	RECIST ICR	Atz (1200 mg) q3w + Cbdca (AUC 6) q3w + nPtx (100 mg/m2) q1w<br>Cbdca (AUC 6) q3w + nPtx (100 mg/m2) q1w	1/0/5

Patho, pathology; NSCLC, non-small cell lung cancer; SQ, squamous cell carcinoma; NSQ, non-squamous cell carcinoma.

Rec, recurrent; Adv, advanced; Met, metastasis; LocAdv, locally advanced;

PD-L1, programmed death ligand 1; TC, tumor cells; IC, tumor-infiltrating immune cells; >1%, 1% or higher; >5%, 5% or higher.

PS, performance status (if not specified, Eastern Cooperative Oncology Group PS).

RECIST, Response Evaluation Criteria in Solid Tumours version 1.1; ICR, independent central review.

Plat, platinum; ATZ, atezolizumab; Cbdca, carboplatin; nPtx, nab-paclitaxel; Ptx, paclitaxel; AUC, area under curve; Bev, bevacizumab. q3w, every 3 weeks.

ROB, Cochrane risk of bias; H/U/L, high/unclear/low risk of bias; NA, not applicable because the study was not an RCT.

Figure S1. Scatter plot of overall survival and progression-free survival using independent-patient-data.

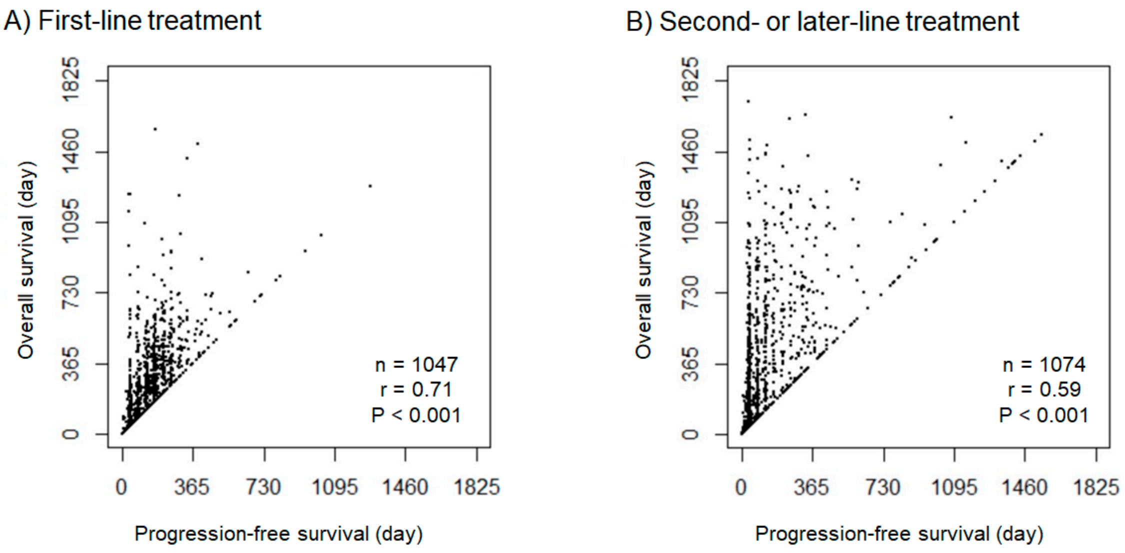

N, number of patients.

r, Spearman's rank correlation coefficient.

Figure S2. Preferred Reporting Items for Systematic Reviews and Meta-Analyses flow diagram for the trial-level analysis.

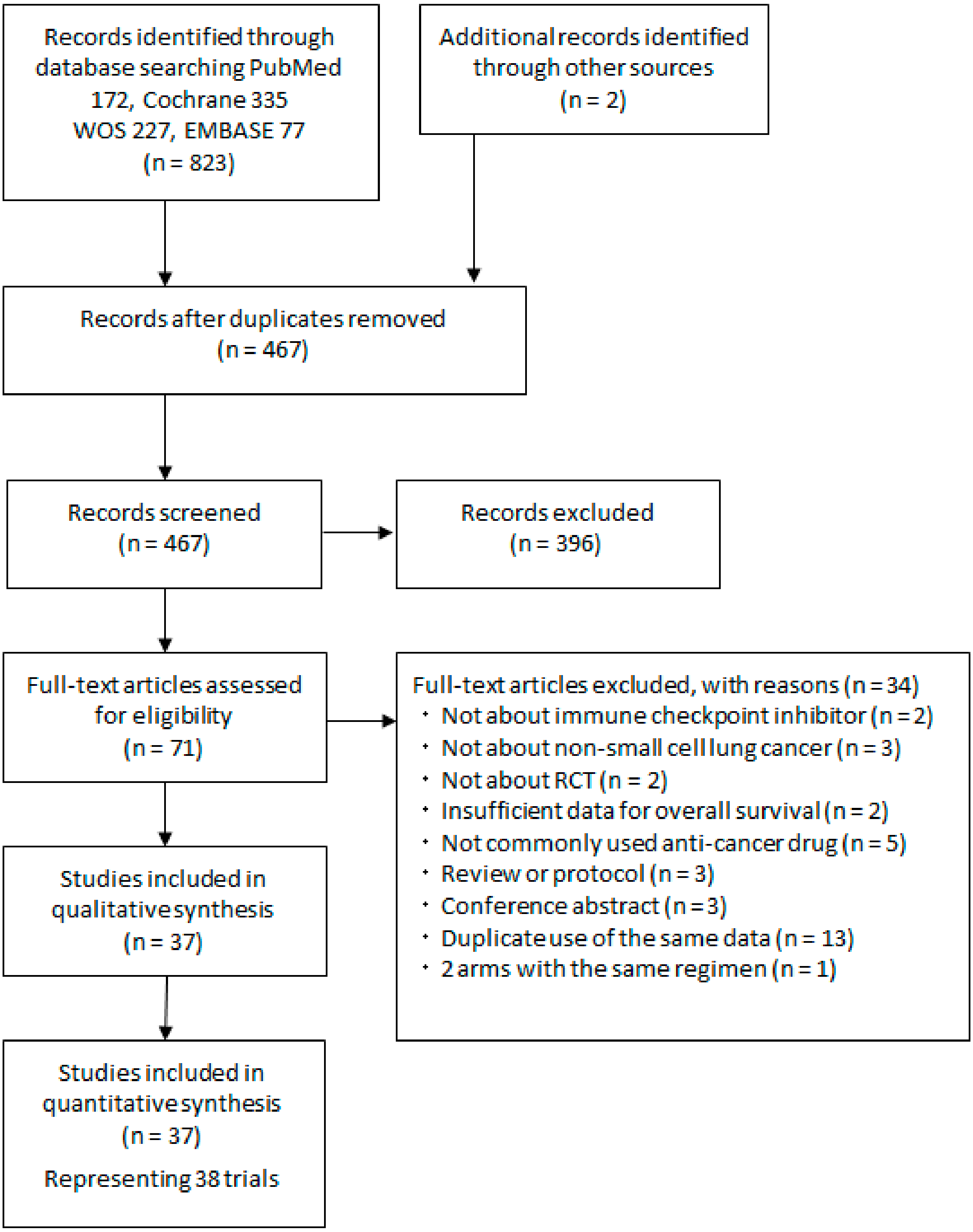

Figure S3. Sample data used for Monte Carlo simulation.

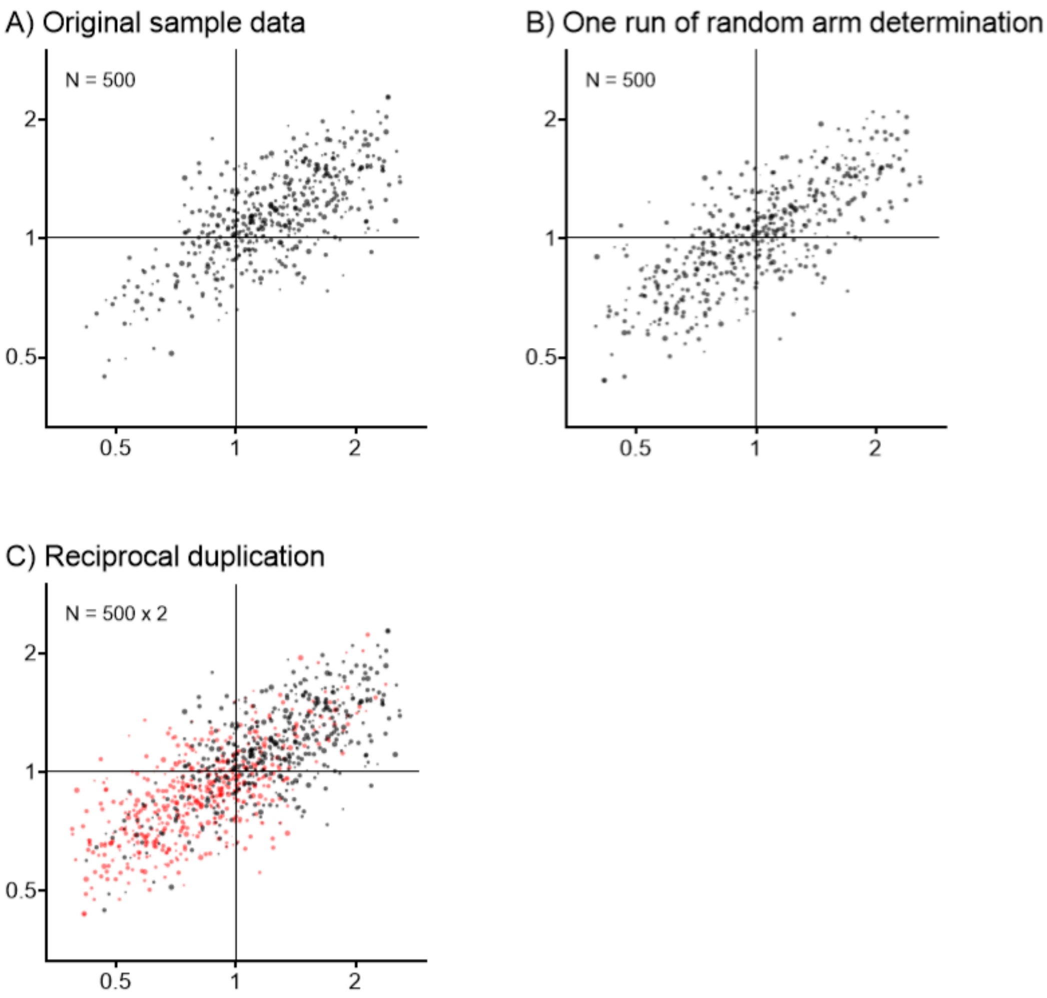

A dot represents a 2-arm trial. Size of a dot indicates weight of a study.

A) Many dots are plotted in right upper quadrant, where suggests x- and y-axes values >1, meaning that more events were observed in the experimental than in the reference arm.

B) This figure illustrates one run of random arm determination with 500 trials. The experimental and standard arms were randomly selected. Similar numbers of dots were equally plotted in the right upper and left lower quadrants, indicating that the overall event frequencies were similar in both arms. Another run of random arm determination with 500 trials is expected to produce a similar result because the sample size is sufficiently

large.

C) Reciprocal duplication. For each of the 500 2-arm trials, original position is plotted in black and the position of point symmetry is plotted in red.

We hypothesized that the correlation coefficient based on random arm determination (B) was an approximation of the correlation coefficient following reciprocal duplication (C).

Figure S4. Monte-Carlo simulation for reciprocal duplication.

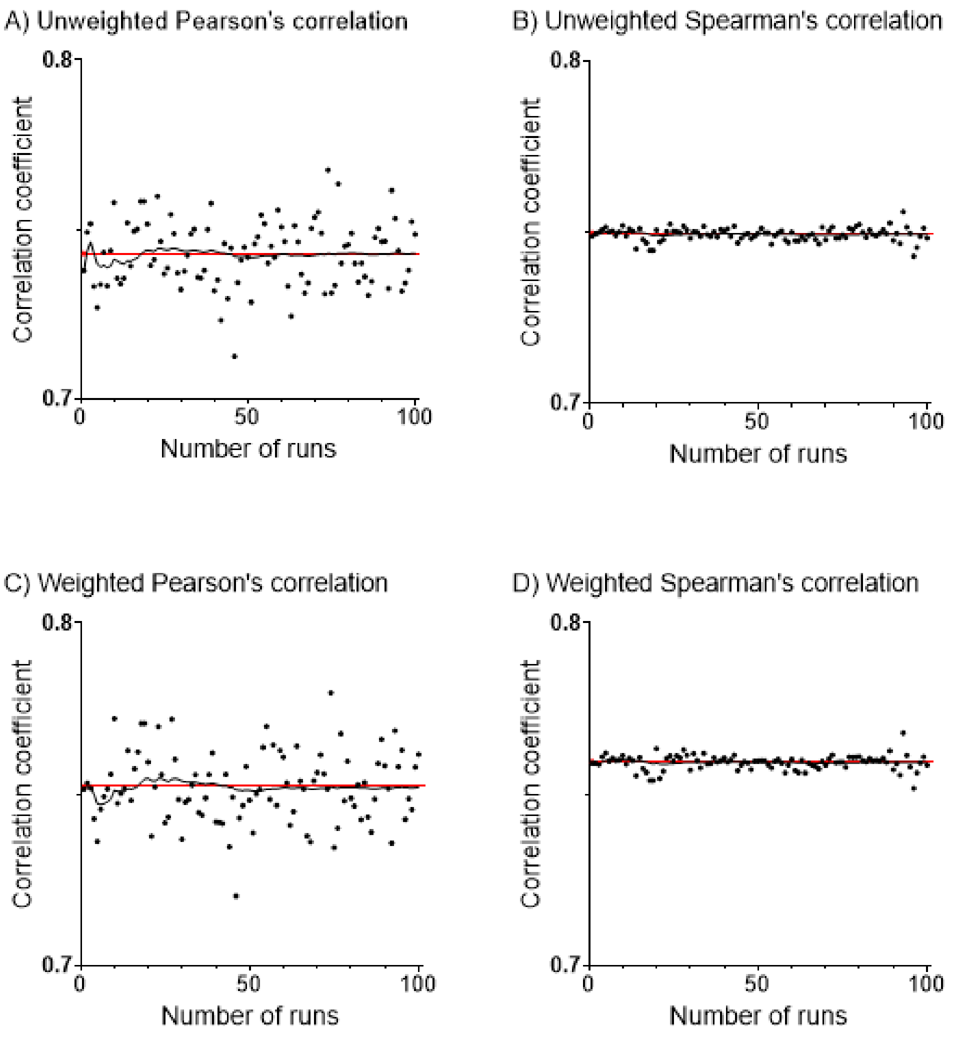

Dot: Each dot represents one run of random arm determination for 500 2-arm trials.

Black line: Average correlation coefficient of present and previous runs.

Red line: Correlation coefficient based on reciprocal duplication ($N = 500 \times 2$).

As the number of runs increased, the black line approached to the red line.

Reference S1. Review articles used in hand search

1. Chen DL, Li QY, Tan QY. Smoking history and the efficacy of immune checkpoint inhibitors in patients with advanced non-small cell lung cancer: a systematic review and meta-analysis. J Thorac Dis. 2021;13(1):220-U123.

2. Chen R, Hou XM, Yang LP, Zhao D. Comparative efficacy and safety of first-line treatments for advanced non-small cell lung cancer with immune checkpoint inhibitors: A systematic review and meta-analysis. Thoracic Cancer. 2019;10(4):607-623.

3. Chen S, Hu B, Li H. A meta-analysis of nivolumab for the treatment of advanced non-small-cell lung cancer. Onco Targets Ther. 2018;11:7691-7697.

4. Chen Y, Zhou Y, Tang L, et al. Immune-Checkpoint Inhibitors as the First Line Treatment of Advanced Non-Small Cell Lung Cancer: A Meta-Analysis of Randomized Controlled Trials. J Cancer. 2019;10(25):6261-6268.

5. Frederickson AM, Arndorfer S, Zhang I, et al. Pembrolizumab plus chemotherapy for first-line treatment of metastatic nonsquamous non-small-cell lung cancer: a network meta-analysis. Immunotherapy. 2019;11(5):407-428.

6. Galvano A, Gristina V, Malapelle U, et al. The prognostic impact of tumor mutational burden (TMB) in the first-line management of advanced non-oncogene addicted non-small-cell lung cancer (NSCLC): a systematic review and meta-analysis of randomized controlled trials. ESMO Open. 2021;6(3):100124.

7. Huang Q, Zhang H, Hai J, et al. Impact of PD-L1 expression, driver mutations and clinical characteristics on survival after anti-PD-1/PD-L1 immunotherapy versus

chemotherapy in non-small-cell lung cancer: A meta-analysis of randomized trials. Oncoimmunology. 2018;7(12):e1396403.

8. Kim BJ, Kim JH, Kim HS. Survival benefit of immune checkpoint inhibitors according to the histology in non-small-cell lung cancer: A meta-analysis and review. Oncotarget. 2017;8(31):51779-51785.

9. Kim J, Ha H, Park J, Cho J, Lim JH, Lee MH. Association of Smoking Status with Efficacy of First-line Immune Checkpoint Inhibitors in Advanced Non-small Cell Lung Cancers: A Systematic Review and Meta-analysis. J Cancer. 2022;13(2):364-372.

10. Kim JH, Kim HS, Kim BJ. Prognostic value of KRAS mutation in advanced non-small-cell lung cancer treated with immune checkpoint inhibitors: A meta-analysis and review. Oncotarget. 2017;8(29):48248-48252.

11. Landre T, Justeau G, Assie JB, et al. Anti-PD-(L)1 for KRAS-mutant advanced non-small-cell lung cancers: a meta-analysis of randomized-controlled trials. Cancer Immunology Immunotherapy.

12. Li DN, Lu WQ, Yang BW, et al. Atezolizumab Monotherapy or Plus Chemotherapy in First-Line Treatment for Advanced Non-Small Cell Lung Cancer Patients: A Meta-Analysis. Front Immunol. 2021;12:666909.

13. Li S, Zhang S, Liu JJ, Yang CL, Zhang L, Cheng Y. The effect of PD-L1/PD-1 immunotherapy in the treatment of squamous non-small-cell lung cancer: a meta-analysis of randomized controlled clinical trials. J Thorac Dis. 2019;11(11):4453-+.

14. Liu TT, Ding SL, Dang J, Wang H, Chen J, Li G. First-line immune checkpoint

inhibitors for advanced non-small cell lung cancer with wild-type epidermal growth factor receptor (EGFR) or anaplastic lymphoma kinase (ALK): a systematic review and network meta-analysis. J Thorac Dis. 2019;11(7):2899-+.

15. Peng S, Ying AF, Tai BC, Soo RA. A meta-analysis on immune checkpoint inhibitor efficacy for advanced non-small cell lung cancer between East Asians versus non-East Asians. Transl Lung Cancer Res. 2020;9(4):1124-1137.

16. Peng TR, Lin HH, Tsai FP, Wu TW. Immune checkpoint inhibitors for first-line treatment of advanced non-small-cell lung cancer: A systematic review and network meta-analysis. Thoracic Cancer. 2021;12(21):2873-2885.

17. Peng TR, Wu TW. Efficacy of PD-1/PD-L1 inhibitors in patients with advanced non-small cell lung cancer: A meta-analysis of randomized clinical trials. Thoracic Cancer. 2019;10(5):1176-1181.

18. Shi YF, Chen W, Li CY, et al. Efficacy and safety of first-line treatments with immune checkpoint inhibitors plus chemotherapy for non-squamous non-small cell lung cancer: a meta-analysis and indirect comparison. Annals of Palliative Medicine. 2021;10(3):2766-2775.

19. Wu D, Duan CY, Wu FF, Chen LY, Chen SZ. Which treatment is preferred for advanced non-small-cell lung cancer with wild-type epidermal growth factor receptor in second-line therapy? A meta-analysis comparing immune checkpoint inhibitor, tyrosine kinase inhibitor and chemotherapy. Oncotarget. 2017;8(39):66491-66503.

20. Wu SY, Wang L, Li W, et al. Comparison between the first-line and second-line immunotherapy drugs in the progression-free survival and overall survival in advanced

non-small cell lung cancer: a systematic review and meta-analysis of randomized controlled trials. Annals of Palliative Medicine. 2021;10(2):1717-1726.

21. Xu Y, Wang Q, Xie J, et al. The Predictive Value of Clinical and Molecular Characteristics or Immunotherapy in Non-Small Cell Lung Cancer: A Meta-Analysis of Randomized Controlled Trials. Front Oncol. 2021;11:732214.

22. Xu YY, Wan B, Chen X, et al. The association of PD-L1 expression with the efficacy of antiPD-1/PD-L1 immunotherapy and survival of non-small cell lung cancer patients: a meta-analysis of randomized controlled trials. Translational Lung Cancer Research. 2019;8(4):413-+.

23. Xu Z, Yi F, Yu D, Xu J, Wei Y, Zhang W. Nivolumab provides improved effectiveness and safety compared with docetaxel as a second-line treatment for advanced non-small cell lung cancer: A systematic review and meta-analysis. Cancer Med. 2019;8(2):629-642.

24. Yin Q, Dai L, Sun R, Ke P, Liu L, Jiang B. Clinical Efficacy of Immune Checkpoint Inhibitors in Non-small-cell Lung Cancer Patients with Liver Metastases: A Network Meta-Analysis of Nine Randomized Controlled Trials. Cancer Res Treat. 2021.

25. You W, Liu M, Miao JD, et al. A Network Meta-analysis Comparing the Efficacy and Safety of Anti-PD-1 with Anti-PD-L1 in Non-small Cell Lung Cancer. J Cancer. 2018;9(7):1200-1206.

26. Zheng SY, Cui HJ, Duan H, et al. The efficacy and safety of immune checkpoint inhibitors in non-small cell lung cancer patients of different age groups: a meta-analysis. Clin Transl Oncol. 2020;22(7):1146-1154.

27. Zhou Y, Chen C, Zhang X, et al. Immune-checkpoint inhibitor plus chemotherapy versus conventional chemotherapy for first-line treatment in advanced non-small cell lung carcinoma: a systematic review and meta-analysis. J Immunother Cancer. 2018;6(1):155.

28. Zhou YX, Zhang YQ, Guo GF, et al. Nivolumab plus ipilimumab versus pembrolizumab as chemotherapy-free, first-line treatment for PD-L1-positive non-small cell lung cancer. Clinical and Translational Medicine. 2020;10(1):107-115.

29. Zhu H, Xie D, Yu Y, et al. KEAP1/NFE2L2 Mutations of Liquid Biopsy as Prognostic Biomarkers in Patients With Advanced Non-Small Cell Lung Cancer: Results From Two Multicenter, Randomized Clinical Trials. Front Oncol. 2021;11:659200.

30. Zhuansun YX, Huang FT, Du YM, Lin L, Chen R, Li JG. Anti-PD-1/PD-L1 antibody versus conventional chemotherapy for previously-treated, advanced non-small-cell lung cancer: a meta-analysis of randomized controlled trials. J Thorac Dis. 2017;9(3):655-665.

Reference S2. Original articles for trials used for the study-level analysis

1. Antonia SJ, Villegas A, Daniel D, et al. Durvalumab after chemoradiotherapy in stage III non-small-cell lung cancer. N Engl J Med 2017; 377(20): 1919-29.

2. Barlesi F, Vansteenkiste J, Spigel D, et al. Avelumab versus docetaxel in patients with platinum-treated advanced non-small-cell lung cancer (JAVELIN Lung 200): an open-label, randomised, phase 3 study. Lancet Oncol 2018; 19(11): 1468-79.

3. Borghaei H, Paz-Ares L, Horn L, et al. Nivolumab versus Docetaxel in Advanced Nonsquamous Non-Small-Cell Lung Cancer. N Engl J Med 2015; 373(17): 1627-39.

4. Boyer M, Şendur MAN, Rodríguez-Abreu D, et al. Pembrolizumab Plus Ipilimumab or Placebo for Metastatic Non-Small-Cell Lung Cancer With PD-L1 Tumor Proportion Score ≥ 50%: randomized, Double-Blind Phase III KEYNOTE-598 Study. J Clin Oncol 2021; 39(21): 2327-38.

5. Brahmer J, Reckamp KL, Baas P, et al. Nivolumab versus Docetaxel in Advanced Squamous-Cell Non-Small-Cell Lung Cancer. N Engl J Med 2015; 373(2): 123-35.

6. Carbone DP, Reck M, Paz-Ares L, et al. First-line nivolumab in stage IV or recurrent non-small-cell lung cancer. N Engl J Med 2017; 376(25): 2415-26.

7. Fehrenbacher L, Spira A, Ballinger M, et al. Atezolizumab versus docetaxel for patients with previously treated non-small-cell lung cancer (POPLAR): a multicentre, open-label, phase 2 randomised controlled trial. Lancet 2016; 387(10030): 1837-46.

8. Gandhi L, Rodríguez-Abreu D, Gadgeel S, et al. Pembrolizumab plus

Chemotherapy in Metastatic Non-Small-Cell Lung Cancer. N Engl J Med 2018; 378(22): 2078-92.

9. Gettinger SN, Redman MW, Bazhenova L, et al. Nivolumab plus Ipilimumab vs Nivolumab for Previously Treated Patients with Stage IV Squamous Cell Lung Cancer: the Lung-MAP S1400I Phase 3 Randomized Clinical Trial. JAMA oncology 2021.

10. Govindan R, Szczesna A, Ahn MJ, et al. Phase III Trial of Ipilimumab Combined With Paclitaxel and Carboplatin in Advanced Squamous Non-Small-Cell Lung Cancer. J Clin Oncol 2017; 35(30): 3449-57.

11. Hellmann MD, Paz-Ares L, Bernabe Caro R, et al. Nivolumab plus Ipilimumab in Advanced Non-Small-Cell Lung Cancer. N Engl J Med 2019; 381(21): 2020-31.

12. Hensing TA, Wang X, Stinchcombe TE, et al. Alliance Foundation Trial 09: A Randomized, Multicenter, Phase 2 Trial Evaluating Two Sequences of Pembrolizumab and Standard Platinum-Based Chemotherapy in Patients With Metastatic NSCLC. JTO Clin Res Rep 2021; 2(8): 100208.

13. Herbst RS, Baas P, Kim DW, et al. Pembrolizumab versus docetaxel for previously treated, PD-L1-positive, advanced non-small-cell lung cancer (KEYNOTE-010): a randomised controlled trial. Lancet (london, england) 2016; 387(10027): 1540-50.

14. Herbst RS, Giaccone G, de Marinis F, et al. Atezolizumab for First-Line Treatment of PD-L1-Selected Patients with NSCLC. N Engl J Med 2020; 383(14): 1328-39.

15. Jotte R, Cappuzzo F, Vynnychenko I, et al. Atezolizumab in Combination With Carboplatin and Nab-Paclitaxel in Advanced Squamous NSCLC (IMpower131): results From a Randomized Phase III Trial. J Thorac Oncol 2020; 15(8): 1351-60.

16. Jung HA, Park S, Choi YL, et al. Continuation of pembrolizumab with additional chemotherapy after progression with PD-1/PD-L1 inhibitor monotherapy in patients with advanced NSCLC. Clin Cancer Res 2022.

17. Langer CJ, Gadgeel SM, Borghaei H, et al. Carboplatin and pemetrexed with or without pembrolizumab for advanced, non-squamous non-small-cell lung cancer: a randomised, phase 2 cohort of the open-label KEYNOTE-021 study. Lancet Oncol 2016; 17(11): 1497-508.

18. Leighl NB, Laurie SA, Goss GD, et al. CCTG BR34: a Randomized Phase 2 Trial of Durvalumab and Tremelimumab With or Without Platinum-Based Chemotherapy in Patients With Metastatic NSCLC. J Thorac Oncol 2021.

19. Lynch TJ, Bondarenko I, Luft A, et al. Ipilimumab in combination with paclitaxel and carboplatin as first-line treatment in stage IIIB/IV non-small-cell lung cancer: results from a randomized, double-blind, multicenter phase II study. J Clin Oncol 2012; 30(17): 2046-54.

20. Mok TSK, Wu YL, Kudaba I, et al. Pembrolizumab versus chemotherapy for previously untreated, PD-L1-expressing, locally advanced or metastatic non-small-cell lung cancer (KEYNOTE-042): a randomised, open-label, controlled, phase 3 trial. Lancet (london, england) 2019; 393(10183): 1819-30.

21. Nishio M, Barlesi F, West H, et al. Atezolizumab Plus Chemotherapy for First-

Line Treatment of Nonsquamous NSCLC: results From the Randomized Phase 3 IMpower132 Trial. J Thorac Oncol 2021; 16(4): 653-64.

22. Paz-Ares L, Luft A, Vicente D, et al. Pembrolizumab plus Chemotherapy for Squamous Non-Small-Cell Lung Cancer. N Engl J Med 2018; 379(21): 2040-51.

23. Paz-Ares L, Ciuleanu TE, Cobo M, et al. First-line nivolumab plus ipilimumab combined with two cycles of chemotherapy in patients with non-small-cell lung cancer (CheckMate 9LA): an international, randomised, open-label, phase 3 trial. The lancet Oncology 2021; 22(2): 198-211.

24. Planchard D, Reinmuth N, Orlov S, et al. ARCTIC: durvalumab with or without tremelimumab as third-line or later treatment of metastatic non-small-cell lung cancer. Annals of oncology : official journal of the european society for medical oncology 2020; 31(5): 609-18.

25. Reck M, Rodriguez-Abreu D, Robinson AG, et al. Pembrolizumab versus Chemotherapy for PD-L1-Positive Non-Small-Cell Lung Cancer. N Engl J Med 2016; 375(19): 1823-33.

26. Rittmeyer A, Barlesi F, Waterkamp D, et al. Atezolizumab versus docetaxel in patients with previously treated non-small-cell lung cancer (OAK): a phase 3, open-label, multicentre randomised controlled trial. Lancet 2017; 389(10066): 255-65.

27. Rizvi NA, Cho BC, Reinmuth N, et al. Durvalumab With or Without Tremelimumab vs Standard Chemotherapy in First-line Treatment of Metastatic Non-Small Cell Lung Cancer: the MYSTIC Phase 3 Randomized Clinical Trial. JAMA oncology 2020; 6(5): 661-74.

28. Sezer A, Kilickap S, Gümüş M, et al. Cemiplimab monotherapy for first-line treatment of advanced non-small-cell lung cancer with PD-L1 of at least 50%: a multicentre, open-label, global, phase 3, randomised, controlled trial. Lancet 2021; 397(10274): 592-604.

29. Socinski MA, Jotte RM, Cappuzzo F, et al. Atezolizumab for First-Line Treatment of Metastatic Nonsquamous NSCLC. N Engl J Med 2018; 378(24): 2288-301.

30. Sugawara S, Lee JS, Kang JH, et al. Nivolumab with carboplatin, paclitaxel, and bevacizumab for first-line treatment of advanced nonsquamous non-small-cell lung cancer. Ann Oncol 2021; 32(9): 1137-47.

31. West H, McCleod M, Hussein M, et al. Atezolizumab in combination with carboplatin plus nab-paclitaxel chemotherapy compared with chemotherapy alone as first-line treatment for metastatic non-squamous non-small-cell lung cancer (IMpower130): a multicentre, randomised, open-label, phase 3 trial. The lancet Oncology 2019; 20(7): 924-37.

32. Wu YL, Lu S, Cheng Y, et al. Nivolumab Versus Docetaxel in a Predominantly Chinese Patient Population With Previously Treated Advanced NSCLC: CheckMate 078 Randomized Phase III Clinical Trial. J Thorac Oncol 2019; 14(5): 867-75.

33. Yang Y, Wang Z, Fang J, et al. Efficacy and Safety of Sintilimab Plus Pemetrexed and Platinum as First-Line Treatment for Locally Advanced or Metastatic Nonsquamous NSCLC: a Randomized, Double-Blind, Phase 3 Study (Oncology pRogram by InnovENT anti-PD-1-11). J Thorac Oncol 2020; 15(10): 1636-46.

34. Zhou CC, Wu L, Fan Y, et al. Sintilimab Plus Platinum and Gemcitabine as First-

Line Treatment for Advanced or Metastatic Squamous NSCLC: Results From a Randomized, Double-Blind, Phase 3 Trial (ORIENT-12). J Thorac Oncol 2021; 16(9): 1501-11.

35. Zhou CC, Wang ZP, Sun YP, et al. Sugemalimab versus placebo, in combination with platinum-based chemotherapy, as first-line treatment of metastatic non-small-cell lung cancer (GEMSTONE-302): interim and final analyses of a double-blind, randomised, phase 3 clinical trial. Lancet Oncol 2022; 23(2): 220-33.

36. Zhou Q, Chen M, Jiang O, et al. Sugemalimab versus placebo after concurrent or sequential chemoradiotherapy in patients with locally advanced, unresectable, stage III non-small-cell lung cancer in China (GEMSTONE-301): interim results of a randomised, double-blind, multicentre, phase 3 trial. Lancet Oncol 2022; 23(2): 209-19.

37. Zhou CC, Chen GY, Huang YC, et al. Camrelizumab plus carboplatin and pemetrexed versus chemotherapy alone in chemotherapy-naive patients with advanced non-squamous non-small-cell lung cancer (CameL): a randomised, open-label, multicentre, phase 3 trial. Lancet Respiratory Medicine 2021; 9(3): 305-14.
